# High-intensity focused ultrasound provides palliation for liver metastasis causing gastric outlet obstruction: case report

**DOI:** 10.1186/2050-5736-1-9

**Published:** 2013-07-01

**Authors:** Michele Rossi, Claudio Raspanti, Ernesto Mazza, Ilario Menchi, Angelo Raffaele De Gaudio, Riccardo Naspetti

**Affiliations:** 1 Surgical Endoscopy Unit, Careggi Academic and Regional Hospital of Florence, Florence 50134, Italy; 2 Interventional Radiology Unit, Careggi Academic and Regional Hospital of Florence, Florence 50134, Italy; 3 Radiology Department, Careggi Academic and Regional Hospital of Florence, Florence 50134, Italy; 4 Department of Anesthesia and Critical Care, Careggi Academic and Regional Hospital of Florence, Florence 50134, Italy

**Keywords:** High-intensity Focused Ultrasound, Liver Metastasis, Metastatic Disease, Gastric Outlet Obstruction, Non-invasive Debulking

## Abstract

**Background:**

Surgery is the standard of care in several oncologic diseases. However, when non-surgical candidates are not suitable for radical treatment, palliation must be achieved at least. High-intensity focused ultrasound uses ultrasound power that can be sharply focused for highly localised application, as it is a completely non-invasive procedure. Its non-invasiveness appears to be of paramount importance in critically ill patients.

**Case description:**

We describe the use of ultrasound-guided high-intensity focused ultrasound for a large liver metastasis from breast cancer causing gastric outlet obstruction in a metastatic disease. The left liver deposit did not allow the stomach to empty due to its large volume, and the patient was unable to eat properly. The tumour was metastatic, resistant to chemotherapy and had a size that contraindicated an ablation percutaneous technique. To improve the patient's quality of life, ultrasound-guided high-intensity focused ultrasound ablation seemed the only and most suitable option. Therefore, a high-intensity focused ultrasound treatment was performed, no complications occurred and the patient's general condition has improved since the early post-procedural period. Three months after treatment, two body mass index points were gained, and the lesion decreased by 72% in volume as detected through multi-detector computed tomography follow-up.

**Discussion and conclusion:**

Quality of life is an unquestionable goal to achieve, and palliation must be achieved while causing as little harm as possible. In this view, debulking surgery and percutaneous ablation technique seemed not appropriate for our patient. Instead, high-intensity focused ultrasound combined several advantages, no lesion size limit and a totally non-invasive treatment. Thus, this technique proved to be a clinically successful procedure, offering better disease control and quality of life. In circumstances where other alternatives clearly seem to fail or are contraindicated, high-intensity focused ultrasound can be used and can provide benefits. We recommend its use and development in several oncologic diseases, not only for therapeutic purposes but also for the improvement of patient's quality of life.

## Background

Surgery is the standard of care for selected patients with solid tumours of the liver, offering the chance of a complete cure by tumour removal. Unfortunately, the majority of patients are unfit for surgical resections because of the sites of their tumour, advanced disease or poor general condition. Clinicians have been trying to develop new standards of care in these circumstances, such as radiofrequency ablation, percutaneous ethanol injection, cryoablation, microwave coagulation, laser-induced interstitial thermotherapy and, finally, high-intensity focused ultrasound (HIFU).

HIFU uses ultrasound power that can be sharply focused for highly localised application, as it is a completely non-invasive procedure. The liver is one of the first areas where focused ultrasounds have been used [1]. Several groups have used ultrasound-guided high-intensity focused ultrasound (USgHIFU) to treat hepatic neoplasms including primary neoplasms [2,3] or secondary deposits [4], regardless of tumour location [5,6].

HIFU non-invasiveness appears to be of paramount importance in critically ill patients at very high risk for surgery. Moreover, in addition to the advantage gained from a curative point of view, HIFU may improve quality of life, reducing or eliminating tumour-related pain and providing a debulking of large neoplastic lesions [7-10]. The case we describe is primarily related to the improvement of quality of life for a large liver metastasis causing gastric outlet obstruction in metastatic disease.

### Case description

A 49-year-old female with previously diagnosed breast cancer was referred to us with metastasis, bilaterally and extensively in the lung, in the left acetabulum and in the liver, primarily in the left lobe and in the right (segments 5 to 8 and segment 8, respectively). Three years previously, she had undergone a wide excision and axillary lymph node dissection for a ductal carcinoma pT3N1M0. After the surgery, she was treated with an adjuvant radiotherapy regimen of 50 GY in 25 fractions over 5 weeks and first line chemotherapy agents, such as anthracyclines, taxanes (docetaxel) and cyclophosphamide. She completed a long course of endocrine therapy with tamoxifen.

Initially, she was started on a monoclonal antibody therapy with bevacizumab. The disease progressed notwithstanding the chemotherapy treatment.

**Figure 1:**
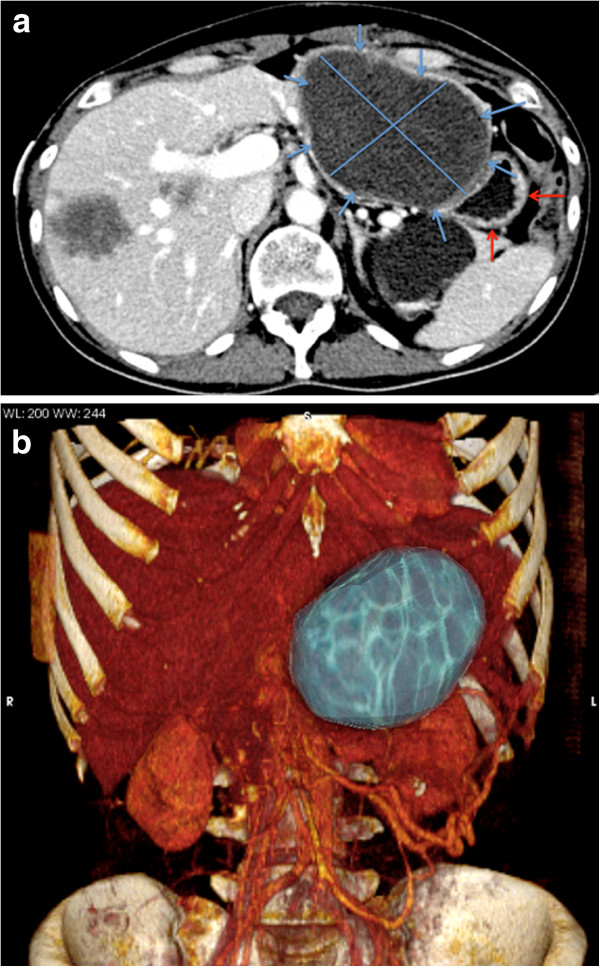
**CT of patient before treatment. (a)** Contrast-enhanced multi-detector CT. A 10 cm × 7 cm liver lesion (blue arrows with main diameters) completely occupying the left lobe in a multi-deposit disease is compressing and dislocating the stomach (red arrows). **(b)** 3D rendering image pre-treatment.

The patient, with a KPS of 70%, presented with anorexia, right upper quadrant pain, weight of loss body mass index (BMI) of 16, vomiting and right coxalgia. A full-body CT scan showed multiple liver and lung deposits, and an osteolytic metastases of the left acetabulum (Figure 1a). A 3D rendering of the pre-HIFU reconstruction clearly showed the extent of the disease (Figure 1b).

The gastric outlet obstruction was due to a left liver metastasis with a volume of 10 cm × 7 cm × 10 cm, corresponding to 365,6993 cm^3^. The lesion volume was calculated by a volumetric semi-automatic segmentation technique analysis on CT scan with slices of 2 mm thin. This lesion caused the most severe symptom upon referral, compressing the duodenum and delaying gastrointestinal transit. Pre-treatment oesophagogastroduodenoscopy confirmed an external obstruction with normal gastric mucosa. To improve patient's quality of life effectively without harm, an HIFU procedure seemed to be the most feasible option for debulking.

### HIFU technique

**Figure 2:**
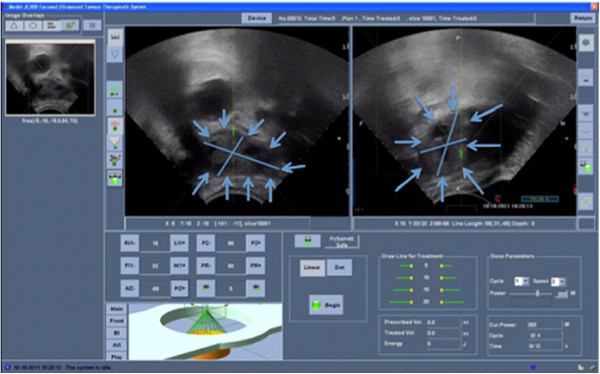
**US pre-treatment images.** Left liver deposit (blue arrows) is in the target area, being completely in the centre of the US scan. There was no gas interference in the acoustic pathway.

Informed consent was obtained from the patient, and approval was gained from Azienda Ospedaliero-Universitaria Careggi local ethics committee. Pre-treatment planning was performed with US, and no gas interference in the acoustic pathway was discovered (Figure 2).

The day before the procedure, bowel preparations had been administered from late morning (MOVIPREP^®^ (Norgine Italia S.r.l., Milan, Italy) - Macrogol 3350/sodium sulphate, anhydrous/sodium chloride/potassium chloride/ascorbic acid/sodium ascorbate plus PANAMIR V (D.M.G. Italia S.r.l. (Rome, Italy) - simethicone), and the patient was put on a full liquid diet. The skin overlying the lesion was carefully shaved to avoid any potential hair interference. General anaesthesia with endotracheal intubation and mechanical ventilation was performed, thus permitting control of the pulmonary inflation. So providing provisional suspension of respiration, it was possible to match the rate of sonication delivery, keeping the target tumour within the therapeutic zone. Nonetheless, the lesion was partially behind the rib cage.

**Figure 3:**
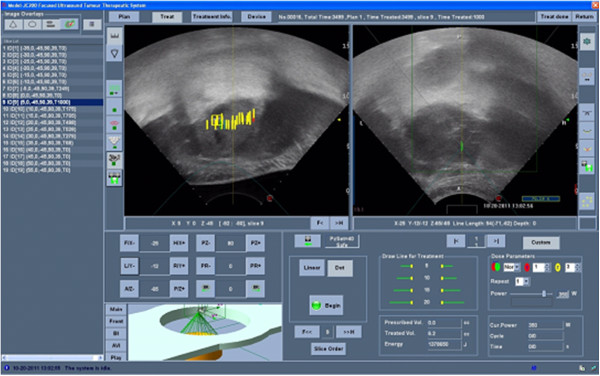
**US images during the treatment.** The yellow lines represent the focal points treated on a single slice. Repeating this process on each slice, we achieve a complete lesion ablation.

A degassed water balloon was used to push and compress the bowel loops to avoid any unexpected presence of air in the beam pathway and to provide bowel-movement control. A nasogastric tube was inserted and left in place to optimise the acoustic pathway and to detect any gastric damage. The patient was carefully positioned in the prone position, placing the skin overlying the target lesion in contact with the degassed water. A vertical scanning mode was chosen with a 5-mm distance between each slice. HIFU ablation was conducted with safe margins of 10 mm within the lesion using the JC200-HIFU system (Chongqing Haifu-HIFU-Tech, Chongqing, China). Therapeutic US energy was produced by a transducer with a diameter of 20 cm, a focal length of 15 cm and a frequency of 0.8 MHz. The MyLab70 US imaging device (ESAOTE, Genova, Italy) was used as a real-time imaging unit, using an integral central 3.5-MHz diagnostic ultrasound probe located in the centre of the HIFU transducer. By scanning with the HIFU beam, the targeted region of each section was ablated. This process was repeated on a section-by-section basis to achieve complete lesion ablation. Real-time US scans showed changes in the echogenicity of the treated regions. Comparing gray-scale changes obtained on the diagnostic images after each HIFU exposure, we assessed and monitored the extent of treatment. If any unexpected residual non-ablated portion of the tumour was found at the site of the treated lesion, an additional HIFU ablation was performed. Total time (preparation and treatment) was 5 h and 30 min. Total treatment time was 3 h and 58 min, with a sonication time of 1 h and 19 min; both of these respective times were related to lesion size and blood supply. The focal peak intensities ranged between 400 and 150 W; however, mainly higher intensity energies were used. The total ablated volume was 80% (Figure 3).

The osteolytic metastasis of the left acetabulum was treated with Cyberknife (Accuray Company, Sunnyvale, USA) stereotactic radiosurgery. Four CT-guided gold fiducials were positioned percutaneously.

## Results

After treatment, the patient started feeding carefully with clear fluid; 3 days later, she was discharged from our hospital with no symptoms remaining and on a semi-liquid diet, with more frequent small meals advised after at least 2 weeks had passed.

The results were considered to be satisfactory based on necrotic tissue formation in the lesion as well as patient clinical signs and symptoms. No ablation-related complications occurred. Laboratory studies showed normal liver function tests 24 h after the procedure.

**Figure 4:**
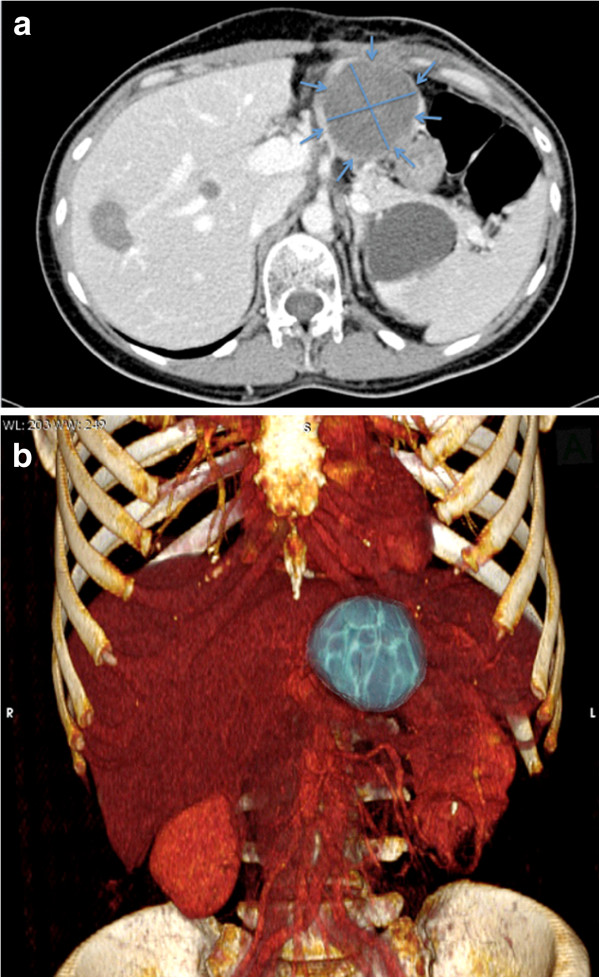
**CT of patient 3 months after treatment. (a)**. Contrast-enhanced multi-detector CT. A 6 cm × 6 cm liver lesion (blue arrows) is identified, showing a decrease in size of 72%. **(b)** 3D rendering image post-treatment.

Three months after the procedure, the patient had a BMI of 18. Two additional points of improvement were shown by post-procedural multi-detector computed tomography (MDCT); the lesion hypodensity with the presence of a residual enhancement rim had decreased in size by approximately 72% (6 cm × 6 cm × 6.5 cm, corresponding to 101.9223 cm^3^), and the lack of enhancement persisted, suggesting successful local tumour control (Figure 4a). A 3D rendering of the post-HIFU reconstruction clearly showed the disease entity (Figure 4b).

## Discussion

The liver is a primary site of metastasis for tumours originating at many sites [11], and liver metastasis develops in approximately half of women with metastatic breast cancer [12]. Therapies for patients with metastatic breast cancer include systemic chemotherapy [13], hormone therapy or both [14]. Immunotherapies, commonly cyclooxygenase-2 or vascular endothelial growth factor inhibitors such as bevacizumab [15,16], are gaining wide acceptance as adjuvant agents in a chemotherapy regimen, but not as a standard treatment.

Our patient unfortunately showed disease progression notwithstanding chemotherapy treatments, such as anthracyclines, taxanes (docetaxel), cyclophosphamide, tamoxifen and monoclonal antibody therapy (bevacizumab).

Quality of life is an unquestionable goal to achieve in these patients. In our experience, we found that the left liver metastasis was the cause of an outlet obstruction syndrome. Palliation must be achieved while causing as little harm as possible. Thus, surgery plays a role if in-depth knowledge of non-surgical palliative treatments is not available. Moreover, surgeons must consider the risk of potentially shortening or reducing the quality of the patient's remaining time due to surgical complications [17,18].

In our experience, HIFU non-invasiveness is of paramount importance. In fact US waves can penetrate skin and tissue layers without causing damage to them, thereby reaching a target further inside the body [19]. At the focal point, the clinical effect occurs by the following two main mechanisms: the thermal effect, wherein temperatures can reach 60°C, with cell death occurring at 56°C for > 1 s [20], and the cavitation-induced cellular damage mechanical effect which is due to mechanical stress [21]. Consequently, the target tissue is destroyed by protein coagulation.

HIFU is gaining increasing interest for the treatment of solid neoplasms [22]. In recent years, many trials have been conducted proving the efficacy and feasibility of HIFU ablation in different clinical applications [23-27]. Liver tumours have been treated when considered as primary neoplasms [2,3] or secondary deposits [4] regardless of tumour location [5,6]. Moreover, in addition to the advantage gained from a curative point of view, HIFU may improve quality of life with uterine fibroids [7], reduce or eliminate tumour-related pain in pancreatic cancer [8,9] or provide debulking of large neoplastic lesions [10].

In our case, a debulking procedure was needed, but the size of the lesion (10 cm × 7 cm × 10 cm) was a contraindication to local ablative therapies, such as radiofrequency ablation [28], percutaneous ethanol injection [29], cryoablation [30], microwave coagulation [31], and laser-induced interstitial thermotherapy [32]. USgHIFU was our last chance to non-invasively treat the liver metastasis. HIFU has been shown to provide ablation of large liver tumours ranging in size from 5 to 12 cm in diameter [33], with the maximum size reached 4 to 14 cm in diameter and an average diameter of 8.18 ± 3.37 cm [34]. Almost the entire lesion was ablated in our case (80% of the lesion), with a decrease in size of approximately 72% at 3 months by MDCT, with the presence of residual enhancement rim. Our goal was a palliative debulking, and this goal was achieved here.

No complications were detected in our case; in fact, outside the focal region, the ultrasound intensity is substantially lower, thus minimising the risk of unintended injury to the surrounding structures. Generally, HIFU is well tolerated, even in severe conditions [35], and this result was confirmed by our case.

## Conclusions

HIFU proved to be a clinically successful procedure, offering better disease control and quality of life. The patient became symptom-free, and no complications occurred. In circumstances where other alternatives clearly seem to fail or are contraindicated, HIFU can be used and provides benefits. We recommend its use and development in several oncologic diseases, not only for therapeutic purposes but also for the improvement of patient's quality of life.

## Abbreviations

HIFU: High-intensity focused ultrasound; USgHIFU: Ultrasound-guided high-intensity focused ultrasound; BMI: Body mass index; MDCT: Multi-detector computed tomography.

## Competing interests

The authors declare that they have no competing interests.

## Authors’ contributions

MR and RN designed the research; CR, EM and IM contributed to the data acquisition and analysis. ARDG contributed to data interpretation. MR, RN and ARDG wrote the paper. All authors read and approved the final manuscript.
